# Increased fibrinolysis-induced bradykinin formation in hereditary angioedema confirmed using stored plasma and biotechnological inhibitors

**DOI:** 10.1186/s13104-019-4335-8

**Published:** 2019-05-27

**Authors:** François Marceau, Hélène Bachelard, Georges-Étienne Rivard, Jacques Hébert

**Affiliations:** 1 Axe Microbiologie-Infectiologie et Immunologie, CHU de Québec-Université Laval, Quebec, QC G1V 4G2 Canada; 2 Axe Endocrinologie et Néphrologie, CHU de Québec-Université Laval, Quebec, QC G1V 4G2 Canada; 3 Division of Hematology/Oncology, CHU Ste-Justine, Montreal, QC H3T 1C5 Canada; 4 Service d’allergie, CHU de Québec-Université Laval, Quebec, QC G1V 4G2 Canada

**Keywords:** Bradykinin, Hereditary angioedema with C1-INH deficiency, Kallikreins, Fibrinolysis, Lanadelumab

## Abstract

**Objective:**

We recently investigated the pathways of immunoreactive bradykinin (iBK) formation in fresh blood of normal volunteers and of patients with hereditary angioedema due to C1-esterase inhibitor deficiency (HAE-1/-2). Herein, we adapted the techniques to small volumes (200 μl) of previously frozen citrated plasma and further analyzed the mechanisms of iBK formation with additional biotechnological inhibitors.

**Results:**

Measurable iBK formation was observed under stimulation with tissue kallikrein (KLK-1, 10 nM), the particulate material Kontact-APTT (concentration reduced to 2% v/v) or recombinant tissue plasminogen activator (tPA, 169 nM), with little background in unstimulated plasma incubated for up to 2 h. Plasma samples from HAE-1/-2 patients responded earlier to tPA than those from controls, as previously reported with whole blood. Lanadelumab inhibited iBK formation induced by Kontact-APTT and tPA. A highly specific plasmin inhibitor, DX-1000, abolished tPA-induced iBK formation in plasma but had no effect against Kontact-APTT, confirming the role of fibrinolysis in tPA-induced kinin formation. The anti-lanadelumab neutralizing antibody M293-D02 reversed the inhibitory effects of lanadelumab. Frozen plasma is a suitable material for measuring iBK formation kinetics, with possible applications such as investigating the effect of rare disease states on the kallikrein–kinin system and monitoring the effect of HAE prophylactic treatments.

**Electronic supplementary material:**

The online version of this article (10.1186/s13104-019-4335-8) contains supplementary material, which is available to authorized users.

## Introduction

We recently investigated the pathways of immunoreactive bradykinin (iBK) formation in fresh blood of normal volunteers and of patients with hereditary angioedema (HAE) due to C1 esterase inhibitor (C1-INH) deficiency (mutations of the SERPING1 gene) [1]. In the case of type 1 and 2 HAE (HAE-1, HAE-2) patients, the blood was sampled during remission. Blood samples were submitted to various standardized forms of in vitro stimulation before extraction. It was found that contact system activation, recombinant tissue kallikrein (KLK-1) or tissue plasminogen activator (tPA) triggered abundant iBK formation, but not the activation of platelets or leukocytes present in the fresh blood. Only tPA was significantly more active in HAE patients, releasing iBK faster and more intensely during the 1st h of incubation [1].

The present work has several goals. Since blood cells do not contribute to kinin formation in a measurable manner, the platform could be more versatile by testing iBK formation in citrated plasma using a scaled down sample volume (200 μl per experimental point) and light benchtop equipment. Also, we decreased the intensity of the activation of contact system with Kontact-APTT to detect possible differences between healthy volunteers and HAE patients. Remaining frozen plasma samples from subsets of previously studied healthy volunteers or HAE patients were exploited to validate these approaches. Additional biotechnological inhibitors, the plasmin inhibitor DX-1000 [2] and the plasma kallikrein active site blocking monoclonal antibody, lanadelumab (DX-2930) [3] were used to analyze the mechanisms of iBK formation triggered by selected stimuli. Lanadelumab and other plasma kallikrein inhibitors are being clinically deployed for prophylaxis of HAE attacks [4–6]: our platform may be applied to monitor the effect of the administration of such inhibitors in HAE patients. Characterizing iBK formation in the genetically heterogeneous HAE patients/families with normal C1-INH levels is also an interesting future goal [7]. Indeed, recently discovered mutations of the F12 and PLG genes [8, 9] beg for a physiopathological analysis of the kallikrein–kinin system. Because HAE caused by a mutation in a gene different from SERPING1 is very rare, updated techniques based on easily shipped frozen plasma will make possible a global recruitment of these patients for the physiopathological analysis of iBK formation.

## Main text

### Materials and methods

#### Human participants

This research project has been approved by the ethics board *Comité d’éthique de la recherche, CHU de Québec*-*Université Laval*, file 2018-3857. Adult healthy human subjects or unrelated HAE-1 or -2 patients were studied, during a remission period for the patients. Subject characteristics are described in Additional file 1: Table S1. Venous blood anticoagulated with sodium citrate was obtained without contact with glass [1]. Both volunteers and patients used in the present study constitute subsets of subjects included in the previous study [1], as the remaining fresh blood had been centrifuged, their plasma frozen on the day of their blood sampling and kept at − 80 °C since then. HAE-1/-2 diagnosis was supported by the measurement of C4 and C1-INH [1] (data reported in Additional file 1: Table S1).

#### Enzyme immunoassay (EIA) of BK

Aliquoted frozen plasma from the previous study [1] was used in all experiment. Each iBK concentration value was derived from 200 μl of thawed citrated plasma transferred to a 1.5 ml Eppendorf conical test tube. Table 1 lists stimulatory and inhibitory substances that have been added to plasma to probe the pathways of iBK formation. All tubes contained the angiotensin converting enzyme (ACE) inhibitor enalaprilat (final concentration 130 nM) to isolate the formation of BK from its rapid hydrolysis by its most important inactivating enzyme [10, 11]. The final concentration of KLK-1 (10 nM) and of tPA (169 nM) used in the present experiments have been used previously with whole blood [1] but represent relatively weaker stimuli in the present study because the recombinant proteins were excluded from the blood volume occupied by blood cells. The concentration of Kontact-APTT (2%) has been greatly decreased vs. the previous study involving fresh blood (20% v/v). The tubes were incubated under rotary agitation (300 rpm) in a pre-equilibrated (37 °C) Thermomixer Compact apparatus with 1.5 ml block (Eppendorf) for 0–120 min. When the desired incubation duration was reached, 1 ml of cold (− 20 °C) absolute ethanol was added to each tube to precipitate/denature proteins. Tubes were allowed to sit for at least 1 h on ice, and then were centrifuged (13,000*g*, 1 min, room temperature, Microfuge 16, Beckman Coulter). The translucent supernatants were transferred in a new set of tubes and completely dried (SpeedVac concentrator). The dried extracts, kept at − 80 °C, were resuspended in 200 μl H_2_O for iBK determination. The BK enzyme immunoassay (EIA) kits (Phoenix Pharmaceuticals, Burlingame, CA) contain an assay buffer that was used to further dilute the reconstituted samples (100- or 1000-fold) before direct application to the EIA, used as directed (duplicate determination) [1].

**Table 1:** Stimuli, potentiator or inhibitors of iBK formation

Stimulus	Final concentration in plasma	Site of action	Source
Recombinant active KLK-1	10 nM	Kininogen cleavage, mostly low molecular weight form	DiaMedica, Inc.
Pacific Hemostasis Kontact-APTT	2% v/v without the calcium supplement	Particulate material that triggers the contact system	ThermoFisher Scientific
Recombinant tPA (alteplase, Cathflow)	169 nM	Plasminogen activation	Roche
Enalaprilat	130 nM	ACE inhibition	Kemprotec Ltd. (Maltby, UK)
Lanadelumab (DX-2930), humanized monoclonal antibody	500 nM	Inhibitor of plasma kallikrein [3]	Shire Intl. GmbH
M293-D02, monoclonal antibody	1 μM	Anti-lanadelumab neutralizing antibody [15]	Shire Intl. GmbH
DX-1000, recombinant Kunitz-type inhibitor	1 μM	Inhibitor of plasmin [2]	Shire Intl. GmbH

#### Data analysis

Values are mean ± standard error of the mean (S.E.M.). In the first part of the study (iBK kinetics as a function of time), single comparisons of value pairs were performed with Mann–Whitney test since the variances significantly differed (Prism 5.0, GraphPad Software Inc., San Diego, CA). In the study of inhibitors, sets of values were compared with the Kruskal–Wallis test (non-parametric ANOVA) followed by Dunn’s multiple comparison test to compare selected pairs of values.

### Results and discussion

#### Effect of established stimulants of iBK formation on samples of blood plasma

**Fig. 1:**
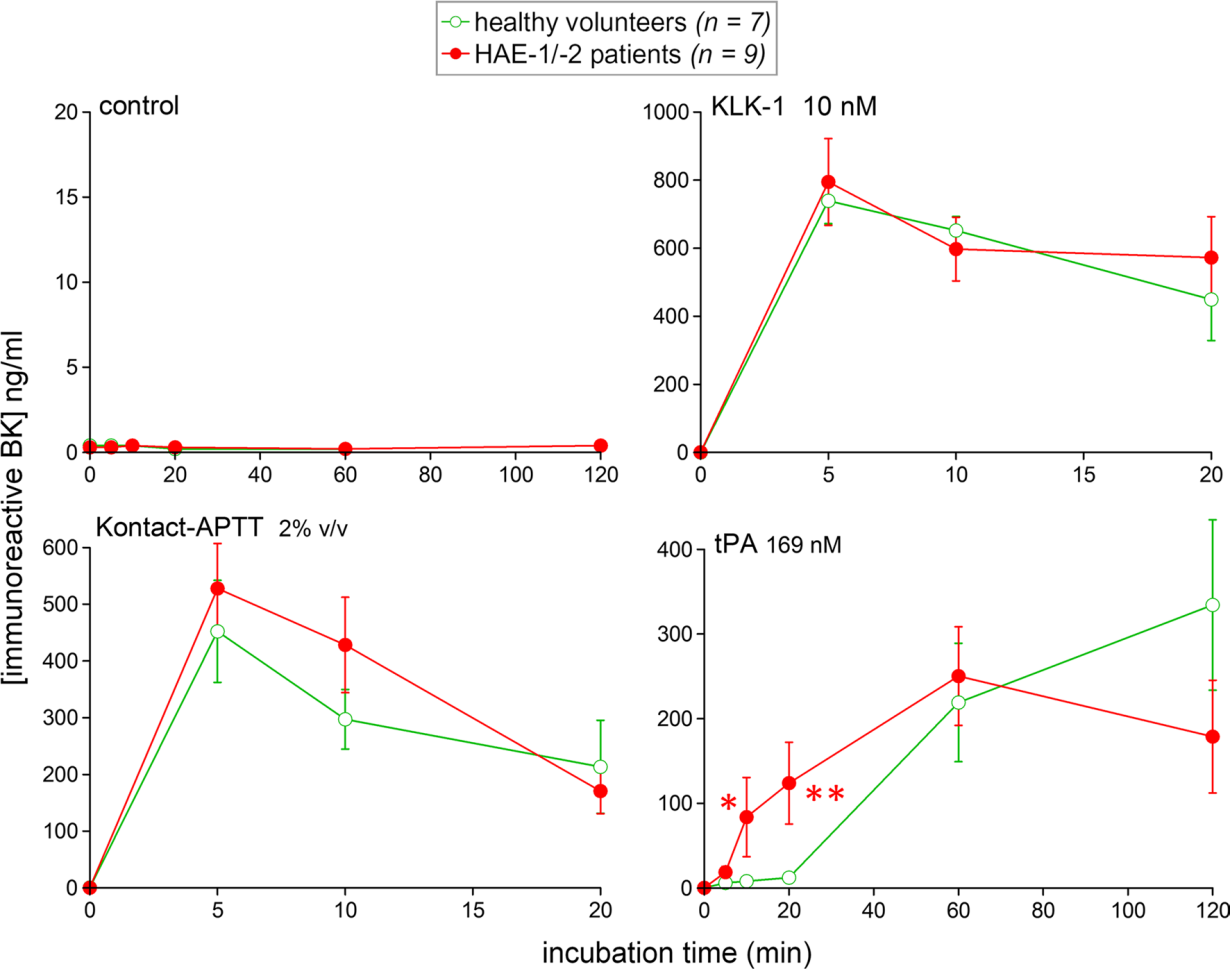
Variation of iBK concentrations as a function of time and stimulation in samples of previously stored citrated blood plasma (incubation at 37 °C in the presence of enalaprilat, 130 nM, added at time zero). Plasma samples originated from healthy subjects or HAE patients with C1-INH deficiency. Values are mean ± S.E.M. for a number of subjects indicated by *n*. For each experimental condition and time point, Mann–Whitney test was applied to isolate the effect of the disease (*P < 0.01; **P < 0.001)

For the first part of the study, the previously frozen plasma from a subset of subjects enrolled in the previous project was submitted to in vitro activation (7 controls and 9 HAE patients; human subjects described in Additional file 1: Table S1). In all reported experiments, the ACE inhibitor enalaprilat was present to reduce iBK breakdown and isolate the kinetics of kinin formation in a more discriminating manner, as previously shown [1]. As reported for whole blood samples [1], iBK remained at background concentrations (≤ 0.4 ng/ml) in control plasma maintained for 0–2 h at 37 °C, whether the samples were from healthy subjects or HAE patients (Fig. 1). Again consistent with findings based on whole blood, iBK concentrations were quickly and persistently increased in response to active KLK-1 (10 nM) in plasma from either type of subjects, with a slow decline in the presence of enalaprilat. The particulate material Kontact-APTT, used as an activator of the contact system, has been added here in plasma in a much diluted manner (final concentration 2% v/v) relative to the concentration previously used in whole blood (20% v/v). However, iBK kinetics remained similar in amplitude vs. those of the previous study, with again no significant effect of the disease (Fig. 1).

tPA retained its very slow kinetics of iBK formation in plasma from healthy volunteers, the first accumulation taking place after 60 min of incubation (Fig. 1). In plasma from HAE patients, a much earlier (≥ 10 min) and initially stronger response was seen, as reported with whole blood [1]. At the time points 10 and 20 min, the disease had a highly significant effect.

#### Effect of novel biotechnological inhibitors

In the previous study based on blood [1], the formation of iBK induced by tPA or Kontact-APTT was abolished by biotechnological inhibitors of plasma kallikrein, either the Kunitz-type peptide inhibitor EPICAL2 [12] or the humanized monoclonal antibody M202-H03 [3]. tPA was further inhibited by the active site inhibitor of factor XII, the monoclonal antibody DX-4012 [13]. KLK-1-induced iBK formation was unaffected by any of these inhibitors, but was virtually abolished by the active site-directed specific inhibitor of that protease, the monoclonal DX-2300 [14].

**Fig. 2:**
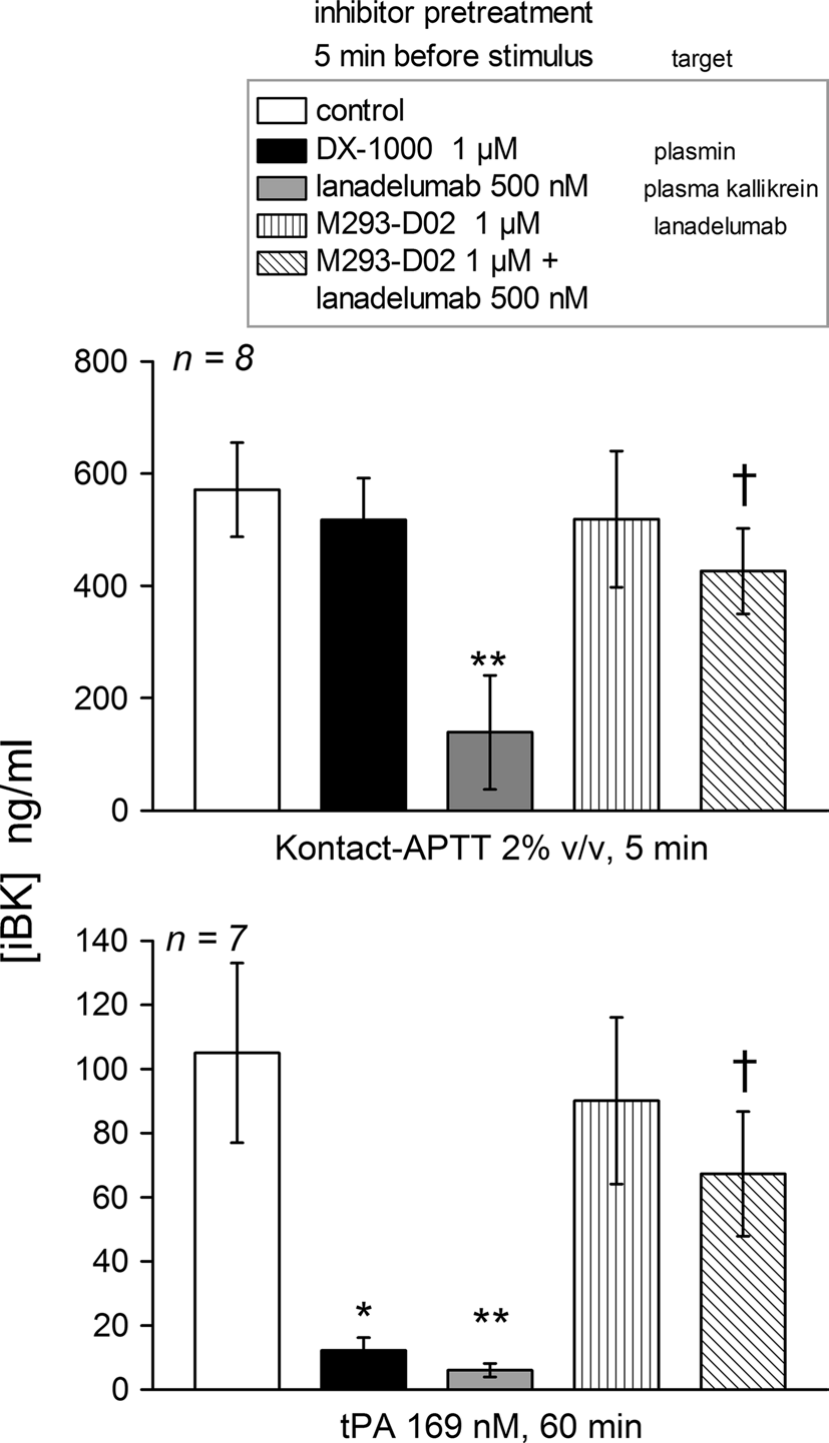
Effect of the plasmin inhibitor DX-1000, the plasma kallikrein inhibitor lanadelumab of the anti-lanadelumab antibody M293-D02 on iBK generation induced by Kontact-APTT or tPA (concentrations, incubation periods as indicated). The inhibitors were added 5 min before the addition of stimuli to plasma samples at time zero. Enalaprilat (130 nM) was present in all tubes. Values are mean ± S.E.M. (number of subjects indicated by *n*). The Kruskal–Wallis ANOVA applied to values of iBK from blood under each of the 2 stimuli indicated significant differences (P = 0.003 for Kontact-APTT, P = 0.0005 for tPA). Selected pairs of values were compared using Dunn’s multiple comparison test. Each value vs. common control: *P < 0.05; **P < 0.01. Effect of lanadelumab alone vs. its combination with M293-D02: ^†^P < 0.05

Additional inhibitors were combined with two activators of iBK formation: the highly selective Kunitz-type plasmin inhibitor, DX-1000 [2] and lanadelumab, a plasma kallikrein inhibitor recently introduced in clinical use for the prevention of HAE attacks [4, 15]. Biotechnological inhibitors were added to plasma samples from 7 or 8 healthy volunteers 5 min before one of the 2 active stimuli was added. The incubation periods selected for Kontact-APTT and tPA were those that produced maximal iBK concentrations (5 and 60 min, respectively) and stimulus concentrations were the same as in the first part of the study (Fig. 1); enalaprilat presence was maintained as well (130 nM). Only lanadelumab significantly reduced iBK formation induced by Kontact-APTT (Fig. 2). Further, the anti-lanadelumab antibody M292-D02 [14], without effect by itself, significantly reversed the inhibition caused by lanadelumab. The same findings applied to tPA-induced iBK formation, except that the plasmin inhibitor DX-1000, without effect against Kontact-APTT, was inhibitory against tPA (Fig. 2). The results confirm that tPA recruits an intermediate step, the activation of plasminogen, to trigger the contact system, probably via factor XII cleavage as previously discussed [1]. The measurement of iBK formation as a function of time may be suitable to investigate the effect of plasma kallikrein inhibitors in the plasma of HAE patients, especially if blood is also sampled before the initiation of the prophylactic treatment for baseline comparison. As the anti-lanadelumab antibody M292-D02 was confirmed to reverse in vitro the inhibitory effect of lanadelumab on iBK formation, M293-D02 could be an additional tool in the monitoring of lanadelumab prophylactic treatment. The platform may be suitable to study the role of BK in other clinical conditions such as HAE with normal C1-INH or acquired forms of angioedema.

Thus, HAE-associated changes in iBK formation were confirmed under the modified experimental conditions based on stored and frozen plasma: hypersensitivity to tPA remains the essential change, with no significant difference concerning direct contact system activation in the plasma of HAE-1 or -2 patients. This suggests that the instability of the kallikrein-kinin system in HAE-1/-2 resides in the upstream fibrinolytic system, as previously discussed [1]. Known triggers of HAE attacks include plausible situations where fibrinolysis may be activated: trauma, infection, medical and dental procedures, menstruation, and, singularly, mental stress [16, 17]. Indeed, mental stress determines tPA release and fibrin turnover in healthy human subjects [18].

## Limitations

In general, the kinetics of iBK formation under the three types of stimulation provide a fully quantitative and rich source of information relative to other methods proposed to study the kallikrein–kinin system in HAE patients, such as the consumption of high molecular weight kininogen with detection of cleavage products that may be based on immunoblotting, a semi-quantitative approach. We have not sampled HAE patients during an attack. Further, most patients received a prophylactic treatment of plasma-derived C1-INH (Berinert), which was not interrupted for the study; any effect on blood chemistry was minimized by sampling the blood at the end of their administration cycle. In fact, the C1-INH levels and C4 consumption were not normalized in the blood of patients under Berinert prophylaxis (Additional file 1: Table S1). The previous study included the corroboration of the presence of a BK-like agonist in extracts of blood samples based on signaling of cultured cells expressing the recombinant B_2_ receptor [1]; this verification was not extended to the present study. KLK-1 is not a part of the contact system and its effect on iBK generation is not modified by HAE-1/-2, but its role is not excluded in other forms of angioedema or anaphylactoid states.

## Additional file

**Additional file 1: Table S1.** Characteristics of human subjects in experiments reported in Fig. 1: patients with HAE with C1-INH deficiency (HAE-1, HAE-2; 6 females, 3 males) or healthy volunteers (5 females, 2 males). For each human subject: age range, diagnosis, approximate frequency of attacks, prophylactic treatment, blood levels of C4 and C1-INH.

## Data Availability

The datasets used in the present study are available from the corresponding author on reasonable request. The biotechnological inhibitors were provided by Shire (now part of the Takeda group of companies) and KLK-1, from DiaMedica Therapeutics. All other reagents are commercially available.

## Abbreviations

ACE: angiotensin converting enzyme
BK: bradykinin
HAE: hereditary angioedema
HAE-1: type 1 HAE
HAE-2: type 2 HAE
iBK: immunoreactive bradykinin
KLK-1: tissue kallikrein
tPA: tissue plasminogen activator
