# Supplementary material for: Increased fibrinolysis-induced bradykinin formation in hereditary angioedema confirmed using stored plasma and biotechnological inhibitors
Source: BMC Res Notes. 2019 May 27;12:291. doi: 10.1186/s13104-019-4335-8 (PMC6537381; doi:10.1186/s13104-019-4335-8)
Supplement: Supplementary file 1 — Additional file 1: Table S1. Characteristics of human subjects in experiments reported in Fig. 1: patients with HAE with C1-INH deficiency (HAE-1, HAE-2; 6 females, 3 males) or healthy volunteers (5 females, 2 males). For each human subject: age range, diagnosis, approximate frequency of attacks, prophylactic treatment, blood levels of C4 and C1-INH. [file 13104_2019_4335_MOESM1_ESM.docx]

**Table S1. Characteristics of human subjects in experiments reported in Fig. 1: patients with HAE with C1-INH deficiency (HAE-1, HAE-2; 6 females, 3 males) or healthy volunteers (5 females, 2 males).***

| **Age range (yr)** | **diagnosis** | **approximate frequency of attacks: every** | **prophylactic treatment** | **C4 (g/l)** | **C1-INH (%)** |
| --- | --- | --- | --- | --- | --- |
| 25-29 | HAE-2 | 6-12 months | none | 0.09 | 14.1 |
| 20-25 | HAE-1 | 4 months | Berinert | 0.12 | 28.5 |
| 25-29 | HAE-1 | 2 weeks | Berinert | 0.15 | 33.7 |
| 60-64 | HAE-1 | 1 month | none | 0.09 | 12.4 |
| 55-59 | HAE-1 | 4 months | Berinert | 0.17 | 38.5 |
| 50-54 | HAE-2 | few days | Berinert | 0.20 | 7.4 |
| 50-54 | HAE-2 | few months | Berinert | 0.14 | 46.9 |
| 15-19 | HAE-1 | few months | Berinert | 0.11 | 11.4 |
| 40-44 | HAE-1 | frequent before prophylaxis | Berinert | 0.10 | 3.9 |
| 60-64 | Healthy | N/A** | N/A | 0.27 | 104.2 |
| 20-24 | Healthy | N/A | N/A | 0.22 | 105.4 |
| 40-44 | Healthy | N/A | N/A | 0.24 | 101. |
| 35-39 | Healthy | N/A | N/A | 0.24 | 92.2 |
| 35-39 | Healthy | N/A | N/A | 0.15 | 79.6 |
| 55-59 | Healthy | N/A | N/A | 0.20 | 88.8 |
| 60-64 | Healthy | N/A | N/A | 0.26 | 69.4 |

* Average C4 concentration: 0.13 ± 0.01 in HAE patients, 0.23 ± 0.01 in healthy subjects; average C1-INH levels: 21.9 ± 5.1 in HAE patients, 91.5 ± 5.1 in healthy subjects. Effect of the pathology: P < 0.001 and 10^-4^, respectively (Student’s t test).

**N/A: not applicable.
